# Cystic Dilatations of the Common Bile Duct in Adults

**DOI:** 10.1155/1996/28924

**Published:** 1996

**Authors:** Gr. Kouraklis, E. Misiakos, A. Glinavou, G. Karatzas, J. Gogas, G. Skalkeas

**Affiliations:** 2nd Department of Propedeutic Surgery Athens University Medical School Athens Greece

## Abstract

Cystic dilatations of the common bile duct are believed to be of congenital etiology with most cases presenting in childhood. During the last 20 years, 10 patients with cystic dilatations of the bile duct were treated in our Department. There were 5 men and 5 women with an age range of 35–81 years. Clinical presentation consisted of right hypohondrial pain, nausea, vomiting and a history of obstructive jaundice. Diagnosis was established by ultrasound, cholangiography and ERCP in most cases. According to the Todani classification system, 5 patients had type I cysts, 4 had type II and one had type III. At the time of surgery, main associated diseases were choledocholithiasis in 3 cases and cholangitis in 2 cases. One patient (type III) underwent endoscopic sphincterotomy; 4 patients underwent internal drainage and 2 of them developed mild cholangitis postoperatively; 5 patients underwent excision of the cyst and a biliary-enteric bypass and developed no main complications. Patients remained in good health during long-term follow-up. We conclude that cyst excision is the treatment ofchoice for adults in order to reduce postoperative morbidity and the potential risk of malignancy.


*HPB Surgery*, 1996, Vol. 10, pp. 91–95
Reprints available directly from the publisher
Photocopying permitted by license only

© 1996 OPA (Overseas Publishers Association)
Amsterdam B.V. Published in The Netherlands
by Harwood Academic Publishers
Printed in Malaysia

### Cystic Dilatations of the Common Bile Duct in Adults

GR. KOURAKLIS, E. MISIAKOS, A. GLINAVOU, G.KARATZAS, J. GOGAS and G. SKALKEAS

2nd Department of Propedeutic Surgery, Athens University Medical School, Athens, Greece

*(Received 25 June 1995)*

Cystic dilatations of the common bile duct are believed to be of congenital etiology with most cases presenting in childhood. During the last 20 years, 10 patients with cystic dilatations of the bile duct were treated in our Department. There were 5 men and 5 women with an age range of 35–81 years. Clinical presentation consisted of right hypohondrial pain, nausea, vomiting and a history of obstructive jaundice. Diagnosis was established by ultrasound, cholangiography and ERCP in most cases. According to the Todani classification system, 5 patients had type I cysts, 4 had type II and one had type III. At the time of surgery, main associated diseases were choledocholithiasis in 3 cases and cholangitis in 2 cases. One patient (type III) underwent endoscopic sphincterotomy; 4 patients underwent internal drainage and 2 of them developed mild cholangitis postoperatively; 5 patients underwent excision of the cyst and a biliary-enteric bypass and developed no main complications. Patients remained in good health during long-term follow-up. We conclude that cyst excision is the treatment of choice for adults in order to reduce postoperative morbidity and the potential risk of malignancy.

KEY WORDS: Billiary cysts choledochal cyst choledochocele congenital bile duct cysts

Cystic dilatations of the common bile duct, the so-called choledochal cysts are rather rare congenital malformations of the pancreatico-biliary system; their incidence ranges from 1 in 13,000 to one in 2 million live births[1]. They are usually a surgical problem of infancy or childhood; however in approximately 20% of cases, diagnosis is established during adulthood[2]. There is a strong predilection for Asians: more than 75% of reported cases come from the Japanese literature[3]. During the last two decades they are found with increasing frequency due mainly to the advent of modern imaging techniques.

There are controversies concerning surgical management of choledochal cysts. Early reports suggested that internal drainage procedures were acceptable surgical treatment; however, long-term follow-up proved that they are followed by a high rate of late complications. Currently, cyst excision with biliary reconstruction has been recognized as the procedure of choice for choledochal cysts[4,5,6].

The present study describes our experience with cystic bile duct dilatations, analyzing the clinical findings, classification, surgical management and long-term results.

### PATIENTS AND METHODS

Between January 1975 and December 1994, 10 adult patients with choledochal cysts were treated at the 2nd Department of Propedecutic Surgery of Athens University Medical School. Sex, age at operation, clinical symptomatology, associated diseases, laboratory, radiographic and histopathological findings, surgical management and postoperative morbidity and mortality were recorded. Choledochal cysts were classified according to the Alonso-Lej classification[7] with

*Correspondence to*: Gregory Kouraklis, MD, FACS 122 Vasilisis Sophias Avenue Athens 11526 Greece Tel. (01)-7772181 Fax (01)-7791456

Todani modification[8], based on radiographic and operative findings.

Follow-up was obtained by review of medical records, follow-up visits and telephone calls to patients. The results were considered good if patients were asymptomatic or had rare mild episodes of cholangitis that were treated conservatively.

**Table 1** Diagnostic imaging methods.

| *Method* | *No. of patients* | *Accuracy (%)* |
|---|---|---|
| Ultrasonography | 4 | 25 |
| Intravenous cholangiography | 2 | 100 |
| Endoscopic retrograde cholangiography | 4 | 100 |
| Computerized tomography | 4 | 50 |
| At operation | 3 | – |

### RESULTS

There were 5 men and 5 women. Their mean age was 63.2 years, with a range from 35 to 81 years. All patients were white Greeks. Five patients had a solitary fusiform extrahepatic cyst (Type I); 4 patients had an extrahepatic supraduodenal diverticulum (Type II) and one patient had a choledochocele (Type III).

Symptoms were typically chronic and intermittent. Recurrent upper abdominal pain, jaundice, fever, nausea and vomiting were the most common findings, usually occuring in combinations. The classic triad of pain, jaundice and a palpable mass was seen in only one case (10%). Two patients came with acute cholangitis due to concomitant choledocholithiasis. Hepatomegaly was present in one patient with cholestatic cirrhosis. Three patients had laboratory evidence of hepatocellular dysfunction and only one patient had mild hyperamylasemia. Seven patients had associated cholelithiasis, choledocholithiasis or cystolithiasis. Calculi were most often bilirubinate and were associated with thick viscous bile and/or sludge.

The standard imaging methods for the diagnosis of biliary tract disease were used in our patients. Ultrasonography was the least accurate method in diagnosing choledochal cysts preopeatively. Only endoscopic retrograde cholangio-pancreatography (ERCP) and intravenous cholangiography clearly defined anatomy of the extrahepatic biliary system. However, diagnosis was not made until operation in 3 cases (Table 1).

Nine patients underwent laparotomy. No patient had undergone a previous operation. Operative cholangiography was carried out for 7 patients. Cyst size ranged from 1 to 4.5 centimeters. A chronic inflammatory reaction was present in 4 cases.

A variety of operations were performed. Four patients with a type I cyst that were treated early in the series had cystoduodenostomy performed. One patient with a type I cyst underwent cyst excision and Roux-en-Y hepaticojejunostomy. Four patients with a type II cyst underwent cyst excision followed by a drainage procedure: choledochoduodenostomy was performed in 3 of them and a Roux-en-Y choledochojejunostomy in one patient. Concurrent cholecystectomy was perfomed in all 9 patients. One patient with a choledochocele (type III cyst) and concomitant choledocholithiasis underwent endoscopic sphincterotomy and stone extraction (Fig. 1). Histopathologic examination of the surgical specimens revealed that the wall of the choledochal cysts was composed almost exclusively of fibrous connective tissue with a patchy collumnar epithelium as a

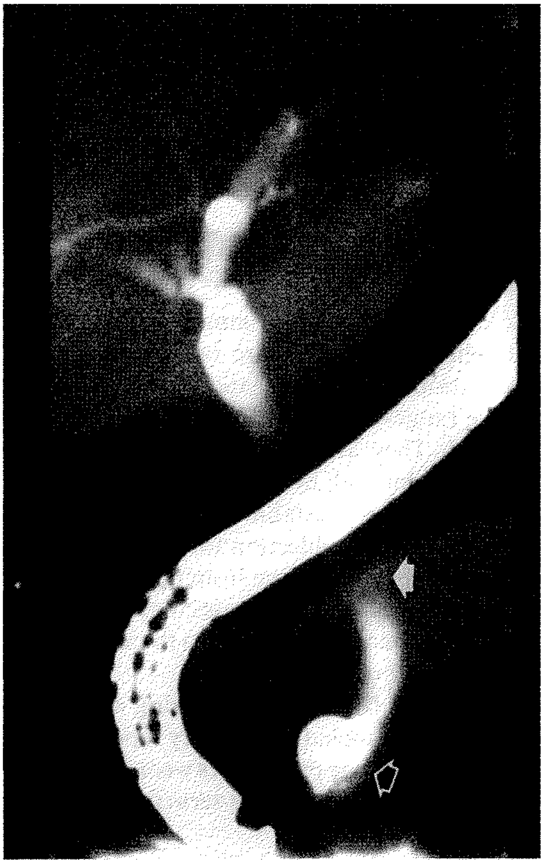

**Figure 1** Endoscopic retrograde cholangio-pancreatography in a patient with choledochocele (open arrow) and concomitant choledocholithiasis (solid arrow).

mucosal lining. Inflammation and mucosal ulceration were present. Chronic cholecystitis was found in 5 patients.

Postoperatively, there was one bile leak that sealed without surgical intervention in a patient undergoing cystoduodenostomy. Two of the early cases treated with internal drainage were lost to long-term follow-up, after an interval of 3 and 4 years during which they did well. At a mean follow-up of 36 months, 2 patients with type I cysts who underwent internal drainage, presented with rare mild episodes of cholangitis that resolved conservatively. One patient had a biopsy proven mild cholestatic cirrhosis two years after operation and is in a good general condition. No patient with a retained cyst presented with malignancy. The patient with a choledochocele treated nonoperatively is doing well 2 years after the endoscopic procedure.

### DISCUSSION

There is still controversy regarding the pathogenesis of choledochal cysts. Yotsuyanagi[9] in 1936 proposed that choledochal cysts arise from inequality in the cellular proliferation of the biliary tract in the early fetal life. Babbitt[10] in 1969 proposed the common channel theory, which is the most widely accepted: patients with cystic bile duct dilatations have an anomalous entry of pancreatic ducts into the bile duct system in such a way that there is a true common channel resulting in easy reflux of pancreatic juice into the duct due to absence of the sphincter of Oddi. In 1959, Alonso-Lej *et al.*[7] developed the first classification system which included type I, II and III choledochal cysts, as are presented in our work. Todani *et al.*[8] in 1977 modified the classification system by adding type IV-A and B and type V cysts. According to previous statistics, type I cysts are reported to make up 85% to 90% of all reported cases worldwide[11], type II cysts occur in less than 2% of cases[4], type III cysts in approximately 2% of cases and type IV cysts occur in 10% of cases[4].

Clinical symptomatology of choledochal cysts varies widely. The classic triad of pain, mass and jaundice described by Alonso-Lej and co-workers is seen in only one third of cases[1,7,12]. In our series most patients had a history of obstructive jaundice where only one patient had a mild hyperamylasemia. The prevalence of hepatobiliary disease, i.e. concurrent lithiasis was greater in our series than usually reported[2,13]; this probably reflects advanced age of our patients and prolonged bile stasis[14].

Between the diagnostic modalities, ultrasonography is considered as one of the best imaging methods for diagnosing choledochal cysts[6]. However in our series its diagnostic accuracy was low. CT scan is useful for revealing associated intrahepatic disease, whereas ERCP and transhepatic cholangiography (PTC) provide the most accurate means for the preoperative examination of the biliary anatomy[1,12].

The conventional surgical approach to choledochal cysts is internal drainage of the cyst into the duodenum or jejunum. This was the procedure we performed in our four early cases of type I cysts. However, longer follow-up revealed that severe late complications such as anastomotic stricture, recurrent cholangitis, lithiasis, pancreatitis, cirrhosis, portal hypertension and carcinoma of the bile duct occured in up to 30–50% of cases[3,4,11,15,16,17]. Kasai was the first to report the increased incidence of bile duct carcinoma arising in cystic bile duct dilatations and proposed surgical excision[18]. Consequently surgical excision and biliary reconstruction is considered now as the procedure of choice. For type I cysts, total excision of the cyst or excision by the method described by Lilly[1,19,20], followed by Roux-en-Y hepaticojejunostomy and cholecystectomy should be performed, as was done in one of our cases. For type II cysts, excision with bile duct repair is indicated, as was done in our cases of type II cysts. Type III cysts are best managed by transduodenal sphincteroplasty or endoscopic sphincterotomy without cyst excision, because of the low risk of carcinoma[3,8,21,22]. This was done in one of four cases of a small type III cyst, using the endoscopic method, and the clinical result was excellent. However an alternative approach by total excision followed by reimlantation of the bile duct and the major pancreatic duct to the duodenal mucosa could be performed[13,23]. No cases of type IV and V cysts occured in our series.

There is still controversy about the management of patients who have undergone internal drainage procedures in the past. There is no doubt that patients with retained cysts carry a lifetime risk of cholangiocarcinoma, which is associated with a dismal prognosis. For that reason, some authors advocate that these patients should undergo a secondary cyst excision[6]. In our series, patients with retained cysts did not developed any severe complications requiring reoperation, except for mild cholangitis in two cases that was managed conservatively and biliary cirrhosis in another case which should rather be attributed to late diagnosis. Further more, no malignancy occured in patients with retained cysts. It is also widely accepted, that excision does not completely eliminate the risk of malignancy, because 40% of cases of cholangiocarcinoma occur outside the cyst[3,6]. We are in accordance with other authors[3,14] who do not recommend reoperation in asymptomatic patients with retained cysts, but meticulous follow-up using biochemical and diagnostic imaging tests, in frequent intervals, during their lifetime.

### REFERENCES

1. Karrer, F. M, Hall, RJ, Stewart, B. A. and Lilly, JR (1990) Congenital biliary tract disease. *Surg Clin North Am*, **70**: 1410–1414.
2. Flanigan, D. P (1975) Biliary cysts. *Ann. Surg.*, **182**: 635–643.
3. Benhidjeb, T., Munster, B., Ridwelski, K., Rudolph, B., Mau, H. and Lippert H (1994) Cystic dilatation of the common bile duct: surgical treatment and long term results. *Br. J. Surg.*, **81**: 433–436.
4. Scudamore, C. H., Hemming, A. W., Teare, J. P., Fache, S, Erb, S. R. and Watkinson, A. F. (1994) Surgical management of choledochal cysts. *Am. J. Surg.*, **167**: 497–500.
5. Lopez, R. R., Pinson, C. W. and Campbell, J. R. (1991) Variation in management based on type of choledochal cyst. *Am. J. Surg.*, **161**: 612–615.
6. Karyal, D., Lees, G. M. (1992) Choledochal cysts, a retrospective review of 28 patients and a review of the literature *Can. J. Surg.*, **35**: 584–588.
7. Alonso-Lej, F., Rever, W. B. Jr., Pessagno, D. J. (1959) Congenital choledochal cysts with a report of 2 and an analysis of 94 cases. *Surg Gynecol Obstet.*, **108**: 1–30.
8. Todani, T., Watanabe, Y. and Narasue, M. (1977) Congenital bile duct cysts. *Am. J. Surg.*, **134**: 263–269.
9. Yotsuyanagi, S. (1936) Contributions to etiology and pathogeny of idiopathic cystic dilatation of the common bile duct with report of three cases: new etiological theory based on supposed inequal epithelial proliferation at the stage of physiological epithelial occlusion of primitive choledochus. *Gann*, **30**: 601–650.
10. Babbitt, D. P. (1969) Congenital cysts; new etiological concepts based on anomalous relationships of the common bile duct and pancreatic bulb. *Ann Radiol*, **12**: 231–240.
11. O'Neil, J. A. (1992) Choledochal cyst. *Curr Probl Surg.*, **29**: 365–410.
12. O'Neil, J. A., Templeton,J. M., Schnaufer L., Bishop, H. C., Ziegler, M. M. and Ross, A. J., (1987) Recent experience with choledochal cyst. *Ann. Surg.*, **205**: 533–540.
13. Dowell, C. S., Sawyers, J. L. and Reynolds, V. H. (1981) Management of adult choledochal cysts. *Ann. Surg.*, **193**: 666–676.
14. Nagorney, D. M., Mc. Ilrath, D. C. and Adson, M. A. (1984) Choledochal cysts in adults: clinical management. *Surgery*, **96**: 656–663.
15. Lilly, J. R. (1979) The surgical treatment of choledochal cyst. *Surg. Gynecol Obstet.*, **149**: 36–42.
16. Trout, H. H. and Longmire, W. P. (1971) Long term follow-up study of patients with congenital cystic dilatation of the common bile duct. *Am. J. Surg.*, **121**: 68–86.
17. Yamaguchi, M. (1980) Congenital choledochal cysts: analysis of 1433 patients in the Japanese literature. *Am. J. Surg.*, **140**: 653–657.
18. Kasai, M., Asakura, Y. and Taira, Y. (1970) Surgical treatment of choledochal cyst. *Ann. Surg.*, **172**: 844–851.
19. Lorenzo, G. A., Seed, R. W. and Beal, J. M. (1971) Congenital dilatation of the biliary tract. *Am. J. Surg.*, **121**: 510–517.
20. Lilley, J. R. (1978) Total excision of choledochal cyst. *Surg., Gynecol, Obstet.*, **146**: 254–256.
21. Martin, R. F., Biber, B. P., Bosco, J. J. and Howell, D. A. (1992) Symptomatic choledochoceles in adults. *Arch. Surg.*, **127**: 536–539.
22. Sarris, G. E. and Tsang, D. (1989) Choledochocele: case report, literature review and a proposed classification. *Surgery*, **105**: 408–414.
23. Bona, S., Boisell, J. C., Harb, J., Descombles, P. H., Rouison, D. and Hugget, C. L. (1989) Development intraduodenal des diverticules du bas choledeque. A propos de deux observations, *Ann Chir.*, **43**: 542–546.

### *Invited Commentary*

Although the typical patient with choledochal cyst has been the young girl (below 10 years of age) presenting with jaundice, abdominal mass and pain, the condition is actually recognised more often in adults[1]. Dr. Kouraklis and colleagues report 10 patients, no less than 4 of whom had type II choledochal cyst. This type makes up no more than 7% in most series[2-4], and it is unusual to find more than 2 cases in any one series.

The authors do not define the entity of choledochal cyst. Only four patients in their series had an endoscopic retrograde cholangiopancreatogram (ERCP), none had percutaneous transhepatic cholangiography (which often gives the best delineation) and two had an intravenous cholangiogram, which generally gives poor definition of bile duct diameter. They are therefore relying upon operative interpretation of bile duct size, which ranged from 1.0 to 4.5 cm. Perhaps this limited investigation explains the apparent lack of any intrahepatic biliary dilatation.

In the present series, ultrasonography was surprisingly poor in diagnosing the cyst, with an accuracy rate of 25 per cent, compared with up to 90% in other series[5,6], even when performed antenatally[7]. Ultrasound scan can sometimes fail to identify extrahepatic biliary dilatation, however, because of the problem with overlying bowel gas[8].

As the authors state, resection of the cyst and bile duct reconstruction is preferable to simple cyst drainage, and their results endorse this fact. Of four patients receiving cyst drainage, two have had recurrent cholangitis and surely need reoperation. The other two are lost to follow-up and must be at some risk of developing cholangiocarcinoma. It is generally agreed that up to 75% of patients need further operation after an internal drainage[2,8,9,10]. In high-risk patients, endoscopic sphincterotomy could be useful if the cause of the recurrent infection were a "sump syndrome" following choledochoduodenostomy[11]; ERCP could also help to show whether the old anastomosis has became stenotic. For patients with type II cyst, excision with bilioenteric anastomosis is usually advocated as the best treatment[3]. Alternatively the cyst can be excised at its junction with the common duct, closing the defect either primarily or with T tube decompression[8].

### BIBLIOGRAPHY

1. Lipsett, P. A., Pitt, H. A., Colombani, P. M., BoinoTT, J. K. and Cameron, J. (1994) Choledochal cyst disease. A changing pattern of presentation. *Ann. Surg.*, **220**: (5) 644–652.
2. Chijiiwa, K. and Koga, A. (1993) Surgical management and long-term follow up of patients with choledochal cysts. *Am. J. Surg.*, **165** (2): 238–242.
3. Lopez, R. R., Pinson, C. V., Campbell, J. R., Harrison, M. and Katon, R. M. (1991) Variation in management based on type of choledochal cyst. *Am. J. Surg.*, **161**(5): 612–615.
4. Hewitt, P. M., Krige, J. E., Bornman, P. C. and Terblanche, J. (1995) Choledochal cysts in adults. *Br. J. Surg.*, **82** (3): 382–5.
5. Akhan, O., Demirkazik, F. B., Ozmen, M. N. and Ariyurek, M. (1994) Choledochal cysts: ultrasonographic findings and correlation with other imaging modalities, *Abdom Imagin* **19**(3): 243–7.
6. Kim, O. H., Chung, H. J. and Choi, B. G. (1995) Imaging of the choledochal cysts. *Radiographics*, **15** (1): 68–69.
7. Howell, C. D., Templeton, J. M., Weirner, S., Glassman, M., Betts, J. M. and Witzleben, C. L. (1983) Antenatal diagnosis and early surgery for choledochal cysts. *J. Pediatr. Surg.*, **18**: 387–393.
8. Buckel, E. G. and Nagorney, D. M. (1994) Choledochal cyst in adult life. Blugmgart, L. H., Surgery of the liver and biliary tract Edinburg, Churchill Livingstone, 1183–1195.
9. Katyal, D. and Lees, G. M. (1992) Choledochal cysts: a retrospective review of 28 patients and a review of the literature. *Can. J. Surg.*, **35** (6): 584–588.
10. Stain, S. C., Guthrie, C. R., Yellin, A. E. and Donovan, A. J. (1995) Choledochal cyst in the adult. *Ann. Surg.*, **222** (2): 128–133.
11. Fan, S. T. and Wong, J. (1994) Recurrent pyogenic cholangitis. Blumgart, L. H., Surgery of the liver and biliary tract. Edinburg. Churchill Livingstone. 1151–1173.

A. M. Isla
R. C. N. Williamson
Department of Surgery Royal Postgraduate Medical School
Hammersmith Hospital, London W12 ONN, UK

